# Comparison of Pulsed Radiofrequency and Endoscopic Piriformis Release for Refractory Piriformis Syndrome: A Propensity Score-Matched Retrospective Cohort Study

**DOI:** 10.3390/jcm14165908

**Published:** 2025-08-21

**Authors:** Eunsung Park, Duyoung Choi, Cheol Lee

**Affiliations:** 1 Department of Neurosurgery, Wonkwang University School of Medicine Hospital, 895 Muwang-ro, Iksan-si 54538, Jeonbuk, Republic of Korea; silverstar0401@gmail.com; 2 Department of Pediatrics, Wonkwang University School of Medicine Hospital, 895 Muwang-ro, Iksan-si 54538, Jeonbuk, Republic of Korea; 3 Department of Anesthesiology and Pain Medicine, Wonkwang University School of Medicine Hospital, 895 Muwang-ro, Iksan-si 54538, Jeonbuk, Republic of Korea

**Keywords:** piriformis syndrome, pulsed radiofrequency, endoscopic piriformis release, sciatic nerve entrapment, pain management, propensity score matching

## Abstract

**Background/Objective:** Piriformis syndrome (PS) causes sciatic nerve entrapment and chronic pain. In refractory cases, pulsed radiofrequency (PRF) and endoscopic piriformis release (EPR) are used, but comparative evidence is limited. **Methods:** This retrospective cohort study compared PRF and EPR in patients treated from 2018 to 2024 at a tertiary hospital using propensity score matching (PSM). Patients with PS, unresponsive to conservative treatment (≥3 months), were included. PRF targeted the sciatic nerve under imaging guidance; EPR involved endoscopic decompression. Primary outcomes were Numeric Rating Scale (NRS) scores at 3 and 6 months. Secondary outcomes included patient satisfaction, reintervention rates, complications, and the Oswestry Disability Index (ODI), where available. After PSM, 115 patients were analyzed per cohort. Multivariate regression identified the predictors of pain improvement. **Results:** From 465 eligible patients (PRF 350; EPR 115), after PSM, 230 patients were analyzed (115 per cohort). The baseline NRS score was 7.4 ± 1.4 (PRF) vs. 7.5 ± 1.3 (EPR). At 3 months, EPR showed a lower NRS score (2.6 ± 1.3) compared to PRF (3.2 ± 1.6; *p* = 0.032). At 6 months, the EPR NRS score was 2.2 ± 1.1 vs. 2.9 ± 1.5 for PRF (*p* = 0.018). EPR had a higher rate of ≥50% NRS score reduction (78% vs. 65%; *p* = 0.041). EPR patients reported higher satisfaction and fewer reinterventions but more complications. Regression analysis identified EPR (OR = 2.15), higher baseline NRS scores, and shorter symptom duration as predictors of improvement. **Conclusions:** EPR provided superior pain relief compared to PRF at 3 and 6 months, although with a higher risk of complications. PRF remains a safer initial option.

## 1. Introduction

Piriformis syndrome (PS) is a neuromuscular disorder characterized by the compression or irritation of the sciatic nerve by the piriformis muscle, resulting in deep gluteal pain, sciatica-like symptoms, and functional limitations. First identified in 1928 by Yeoman, PS accounts for approximately 6–8% of all sciatica cases and is often underdiagnosed because its symptoms overlap with those of other conditions, such as lumbar disk herniation, sacroiliac joint dysfunction, or trochanteric bursitis [1,2,3]. The piriformis muscle, which originates from the anterior surface of the sacrum and attaches to the greater trochanter of the femur, plays an important role in hip external rotation and abduction. Anatomical variations, such as the sciatic nerve passing through or above the piriformis muscle in about 17–20% of people, predispose some patients to entrapment, especially after trauma, overuse, or inflammation [4,5]. Refractory PS was defined as persistent symptoms despite ≥3 months of conservative management, including physical therapy, NSAIDs, muscle relaxants, and at least one piriformis injection. Diagnostic overlap with lumbar and peri-hip pathologies challenges clinical recognition. The validity of PS as a diagnosis is supported by clinical provocation tests and imaging, though it falls under the broader category of deep gluteal syndrome, which encompasses various causes of sciatic nerve entrapment in the subgluteal space [6].

The prevalence of PS is estimated to be higher in females (with a female-to-male ratio of 6:1) and in middle-aged adults, often worsened by sedentary lifestyles, repetitive hip movements in sports, or occupational factors [2,7]. Diagnosis remains difficult, relying on clinical signs like buttock tenderness, positive provocation tests (e.g., Freiberg’s sign, Pace’s sign, or the Flexion, Adduction, and Internal Rotation—FAIR—test), and imaging techniques such as magnetic resonance imaging (MRI) or ultrasound to rule out other conditions and confirm muscle hypertrophy or nerve compression [8,9]. Conservative treatments, including physical therapy, non-steroidal anti-inflammatory drugs (NSAIDs), muscle relaxants, and local injections (e.g., corticosteroids or botulinum toxin), resolve symptoms in 70–80% of cases. However, up to 20% of patients develop refractory PS, defined as persistent pain despite ≥3 months of non-invasive treatment, which requires advanced interventions [1,2,4,5].

Pulsed radiofrequency (PRF) is a minimally invasive neuromodulatory procedure that delivers high-frequency electrical pulses to the sciatic nerve without causing thermal damage, aiming to disrupt pain signaling pathways through electric field effects on neuronal membranes [2,4,7]. In contrast, endoscopic piriformis release (EPR) is a surgical technique that directly decompresses the sciatic nerve by sectioning parts of the piriformis tendon endoscopically, addressing the underlying anatomical compression [10,11]. While both methods have shown effectiveness in small case series—PRF achieving 50–70% pain reduction and EPR up to 80–90%—comparative studies are limited, often characterized by small sample sizes, lack of matching, and short follow-up periods [4,11,12]. This gap in evidence hinders informed decision-making, particularly in determining which patients will benefit most from each approach.

We hypothesized that EPR, by mechanically relieving nerve entrapment, would provide better long-term pain relief compared to PRF’s neuromodulatory effects, although with a potential increase in procedural risks. This study aimed to fill this knowledge gap through a propensity score-matched retrospective cohort analysis, comparing the effectiveness, safety, and success predictors for PRF and EPR in refractory PS. By offering robust, matched data, we intend to guide clinical practice, improve patient selection, and inform future research directions, such as combining regenerative therapies or using advanced imaging for personalized treatment.

## 2. Materials and Methods

### 2.1. Study Design and Population

This retrospective observational cohort study was conducted at the Wonkwang University School of Medicine Hospital, a tertiary care center in Iksan-si, Republic of Korea, from January 2018 to December 2024. The study followed the principles outlined in the Declaration of Helsinki and received approval from the Institutional Review Board of the Wonkwang University School of Medicine Hospital (protocol code: WKUH 2025-03-02-004, approved on 3 March 2025). Because of its retrospective nature, informed consent was waived by the ethics committee.

Patients were identified through electronic medical records using ICD-10 codes for PS (G57.0) and related sciatic neuropathies. Inclusion criteria included adults (≥18 years) diagnosed with PS based on clinical criteria (persistent buttock pain radiating to the leg, positive FAIR test with symptom reproduction, and absence of lumbar radiculopathy on electromyography if indicated) and confirmatory imaging (MRI showing piriformis hypertrophy or sciatic nerve signal changes or ultrasound demonstrating muscle asymmetry) [8,9]. All patients had failed conservative management for at least 3 months, including physical therapy (e.g., stretching, strengthening exercises), NSAIDs, muscle relaxants, and at least one local injection (corticosteroid or botulinum toxin). Interventions were either PRF or EPR, selected based on clinician preference, patient anatomy (e.g., confirmed entrapment on imaging, which favored EPR), and shared decision-making. Refractory PS was defined as persistent symptoms despite ≥3 months of conservative management, including physical therapy, NSAIDs, muscle relaxants, and at least one piriformis injection.

**Figure 1:**
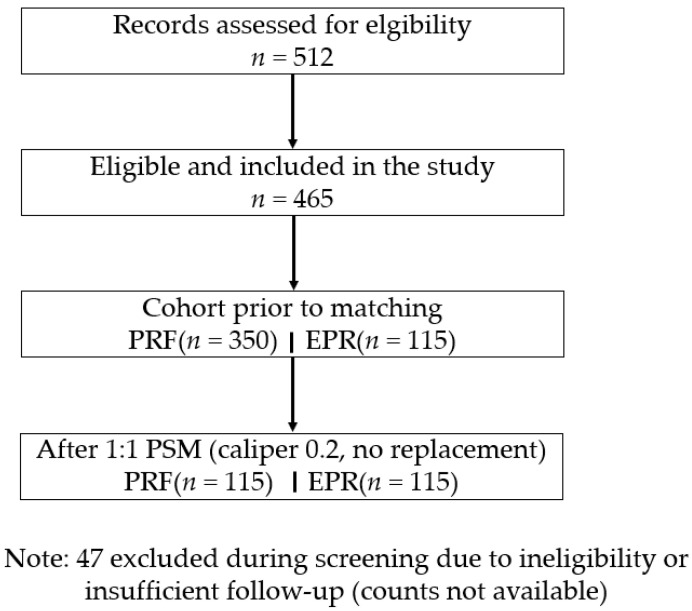
The study flow diagram.

Exclusion criteria included prior lumbar or hip surgery, concurrent spinal pathology (e.g., herniated disk or stenosis confirmed by MRI), systemic neuropathies (e.g., diabetic neuropathy), malignancy, pregnancy, or incomplete follow-up data (<6 months). From an initial pool of 512 records, 465 patients met the criteria (PRF: *n* = 350; EPR: *n* = 115). The main reasons for exclusion were concurrent spinal pathology (e.g., lumbar disk herniation; *n* = 25), incomplete follow-up (*n* = 15), and prior surgery (*n* = 7). See Figure 1 for the study flow diagram.

### 2.2. Interventions

#### 2.2.1. Pulsed Radiofrequency (PRF)

PRF was performed in an outpatient setting under local anesthesia with ultrasound or fluoroscopic guidance. Patients were positioned prone, and the sciatic nerve was localized at the infrapiriformis space using a 22-gauge, 10 cm insulated needle with a 5 mm active tip. Sensory stimulation (50 Hz, 0.5 V) confirmed proximity to the nerve, ensuring paresthesia in the sciatic distribution without motor activation. PRF was delivered at 42 °C for 120 s per cycle, with two cycles applied (total duration: 4 min) using a radiofrequency generator (e.g., Cosman G4). Post-procedure, patients received a steroid injection (e.g., 40 mg triamcinolone) for anti-inflammatory effects and were monitored for 30 min before discharge. All PRF-cohort patients were referred to a standardized post-procedure physical therapy (PT) program within one week (hip strengthening, stretching, and sciatic nerve gliding; see Section 2.2.3).

#### 2.2.2. Endoscopic Piriformis Release (EPR)

EPR was conducted in an operating room under general anesthesia with patients in the lateral decubitus position. A posterior endoscopic approach was utilized, involving two portals, namely a proximal viewing portal at the greater trochanter and a distal working portal in the gluteal fold. Under arthroscopic visualization (30-degree scope), the piriformis tendon was identified, and the sciatic nerve was decompressed by partial tenotomy (releasing 50–70% of the tendon to avoid instability) using electrocautery or scissors. Hemostasis was ensured, and the portals were closed with sutures. Surgery duration averaged 45–60 min. Postoperatively, patients were mobilized the same day, with physical therapy initiated within 48 h, focusing on hip strengthening and nerve gliding exercises. Standardized PT was initiated within 48 h post-op using the same protocol as for PRF. Standardized PT was initiated within 48 h post-op using the same protocol (see Section 2.2.3).

#### 2.2.3. Standardized Physical Therapy Program

Both cohorts followed an identical PT program consisting of 8–12 sessions over 4 weeks, emphasizing progressive hip external-rotator strengthening (e.g., clamshells, side-lying leg lifts), piriformis stretching, and sciatic nerve gliding exercises. Analgesics were prescribed as needed.

### 2.3. Outcomes

The primary outcome was the change in Numeric Rating Scale (NRS) scores (0–10, where 0 indicates no pain and 10 indicates the worst imaginable pain) for buttock/leg pain at 3 and 6 months after the intervention. These were assessed during follow-up visits or by telephone if an in-person visit was not possible. Clinically significant improvement was defined as a ≥50% reduction in NRS scores at 6 months, in line with established thresholds for chronic pain [10,11,13].

Secondary outcomes included

- Patient satisfaction, measured on a 5-point Likert scale (1 = very dissatisfied, 5 = very satisfied), with ≥4 considered satisfactory.
- Reintervention rate within 6 months (e.g., repeat procedure, alternative therapy, or surgery).
- Procedure-related complications (e.g., infection, hematoma, nerve injury, classified as minor or major as per Clavien–Dindo criteria).
- Oswestry Disability Index (ODI) scores (0–100%, higher indicating greater disability) at baseline and 6 months, analyzed in a subgroup with complete data (*n* = 90) as an exploratory endpoint [2,4,7]. Baseline ODI data were incomplete for the full cohort, limiting its inclusion in PSM; subset values are reported in Table 1 and Table 2.

### 2.4. Propensity Score Matching and Balance Assessment

We estimated propensity scores using logistic regression including age, sex, BMI, symptom duration, baseline NRS score, diabetes, and hypertension, with 1:1 nearest-neighbor matching without replacement (caliper 0.2 SD of the logit). Propensity scores were estimated using logistic regression in R (version 4.3.1) with the MatchIt package (4.7.2). Covariate balance was evaluated using standardized mean differences (SMDs) before and after matching, with SMD < 0.10 considered acceptable. A Love plot of SMDs is provided in Supplementary Materials Figure S1.

### 2.5. Statistical Analysis

The normality of continuous variables was assessed using the Shapiro–Wilk test. Normally distributed data were analyzed using paired *t*-tests, while non-normal data were analyzed with Wilcoxon signed-rank tests. Categorical variables were compared using chi-squared or Fisher’s exact tests. The time to reintervention was assessed using Kaplan–Meier survival analysis and log-rank tests. Multivariate logistic regression identified predictors of a ≥50% reduction in NRS scores at 6 months, adjusting for age, sex, BMI, symptom duration, baseline NRS score, and comorbidities. Correlation analysis used Pearson’s (for continuous variables) or Spearman’s (for ordinal/categorical variables) coefficients. Confidence intervals (95% CI) were reported for odds ratios and mean differences. A sample size calculation was targeted to achieve 80% power to detect a 0.5-point NRS score difference (SD = 1.5, α = 0.05), requiring 115 patients per group. *p*-values were two-tailed, expressed to three decimal places (e.g., *p* < 0.001). Analyses were performed using SPSS version 29 (IBM Corp., Armonk, NY, USA).

## 3. Results

### 3.1. Baseline Characteristics

From 512 screened records, 465 met eligibility (PRF 350; EPR 115), and after PSM, 230 patients were analyzed (115 per cohort) (Figure 1).

Pre-matching imbalances (e.g., slightly higher baseline NRS scores in EPR) were resolved post-matching (all *p* > 0.05). Matched cohorts had mean ages of 52.8 ± 11.9 years (PRF) and 53.4 ± 12.2 years (EPR), with 58.3% females in both. BMI was identical at 26.5 ± 4.2 kg/m^2^. Symptom duration averaged 18.6 ± 8.5 months (PRF) vs. 18.4 ± 8.2 months (EPR), baseline NRS scores were 7.4 ± 1.4 vs. 7.5 ± 1.3, diabetes was 14.8% vs. 15.7%, and hypertension was 23.5% vs. 24.3% (Table 1).

**Table 1:** Baseline characteristics before and after propensity score matching.

	Before PSM			After PSM		
Variable	PRF (*n* = 350)	EPR (*n* = 115)	*p*-Value	PRF (*n* = 115)	EPR (*n* = 115)	*p*-Value
Age (years)	51.9 ± 12.3	53.4 ± 12.2	0.231	52.8 ± 11.9	53.4 ± 12.2	0.672
Sex (M/F)	142/208 (59.4% F)	49/66 (57.4% F)	0.701	48/67 (58.3% F)	49/66 (57.4% F)	0.881
BMI (kg/m^2^)	26.2 ± 4.1	26.5 ± 4.2	0.184	26.5 ± 4.2	26.5 ± 4.2	1.000
Symptom duration (months)	19.1 ± 8.7	18.4 ± 8.2	0.435	18.6 ± 8.5	18.4 ± 8.2	0.842
Baseline NRS	7.3 ± 1.4	7.5 ± 1.3	0.199	7.4 ± 1.4	7.5 ± 1.3	0.586
Diabetes (%)	14.3	15.7	0.698	14.8	15.7	0.851
Hypertension (%)	22.6	24.3	0.696	23.5	24.3	0.879

Data are mean ± SD or *n* (%). PSM: propensity score matching; NRS: Numeric Rating Scale; BMI: body mass index. PSM adjusted for age, sex, BMI, symptom duration, and comorbidities. Satisfaction was defined as a 5-point Likert score ≥4. Significant pain reduction was defined as ≥50% reduction in NRS scores.

#### 3.1.1. Primary Outcome

Both groups exhibited significant reductions in NRS scores from baseline (*p* < 0.001 within-group). At 3 months, EPR achieved lower NRS scores (2.6 ± 1.3) than PRF (3.2 ± 1.6; mean difference = 0.6, 95% CI 0.05–1.15, *p* = 0.032). At 6 months, the EPR NRS score was 2.2 ± 1.1 vs. 2.9 ± 1.5 for PRF (mean difference = 0.7, 95% CI 0.12–1.28, *p* = 0.018). The rate of ≥50% NRS score reduction at 6 months was higher in EPR (78%, 90/115) vs. PRF (65%, 75/115; *p* = 0.041) (Table 2) 

#### 3.1.2. Secondary Outcomes

Satisfaction (Likert ≥ 4) was 82% (94/115) in PRF vs. 90% (104/115) in EPR (*p* = 0.092). Complications were more frequent in EPR (7.8%, 9/115: three infections, two nerve irritations, four minor hematomas) than PRF (1.7%, 2/115: two minor hematomas; *p* = 0.053), all minor and resolved conservatively. Reintervention rates were 12% (14/115) in PRF vs. 6% (7/115) in EPR (*p* = 0.134). In the ODI subgroup (*n* = 90), improvements were similar: PRF from 42.3 ± 12.1 to 28.5 ± 10.4 and EPR from 43.1 ± 11.8 to 26.2 ± 9.7 (between-group *p* = 0.198). Complications in PRF included transient paresthesia (*n* = 2); in EPR, minor wound infections (*n* = 4), hematoma (*n* = 3), and temporary nerve irritation (*n* = 2). All resolved without intervention. There were no significant differences in ODI baseline, ODI at 6 months, and ODI change between the two cohorts (Table 2).

**Table 2:** Primary and secondary outcomes after propensity score matching (*n* = 230).

Variable	PRF (*n* = 115)	EPR (*n* = 115)	*p*-Value	Note
NRS at 3 months	3.2 ± 1.6	2.6 ± 1.3	0.032	Primary outcome (difference −0.6, 95% CI −1.1 to −0.1)
NRS at 6 months	2.9 ± 1.5	2.2 ± 1.1	0.018	Primary outcome (difference −0.7, 95% CI −1.1 to −0.3)
≥50% NRS reduction at 6 months (%)	65 (75/115)	78 (90/115)	0.041	OR 1.92, 95% CI 1.03–3.57
Satisfaction (Likert ≥ 4) (%)	82 (94/115)	90 (104/115)	0.092	
Complications (%)	1.7 (2/115)	7.8 (9/115)	0.053	
Reintervention rate (%)	12 (14/115)	6 (7/115)	0.134	
ODI baseline (subset)	42.3 ± 12.1	43.1 ± 11.8	0.71	(*n* = 90)
ODI at 6 months (subset)	28.5 ± 10.4	26.2 ± 9.7	0.29	(*n* = 90)
ODI change (subset)	−13.8 ± 8.2	−16.9 ± 7.5	0.198	(*n* = 90)

Data are mean ± SD or % (*n*). NRS: Numeric Rating Scale; ODI: Oswestry Disability Index.

### 3.2. Time to Reinnervation

**Figure 2:**
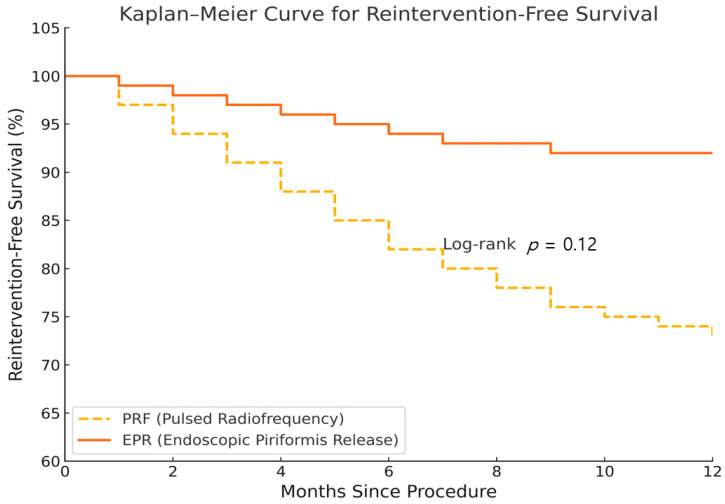
Kaplan–Meier curve for reintervention-free survival.

Kaplan–Meier survival analysis for reintervention showed a non-significant trend favoring EPR (*p* = 0.12). Although the difference did not reach statistical significance, the median time to reintervention was not reached in either group, supporting a longer efficacy duration in EPR (Figure 2).

### 3.3. Multivariate Logistic Regression

Multivariate logistic regression identified predictors of ≥ 50% NRS score reduction at 6 months, with intervention type (EPR vs. PRF: OR = 2.15, 95% CI 1.12–4.13, *p* = 0.021), baseline NRS scores (OR = 1.32 per unit, 95% CI 1.08–1.61, *p* = 0.007), and symptom duration (OR = 0.87 per year, 95% CI 0.76–0.99, *p* = 0.038). Age, sex, BMI, and comorbidities were not significant (*p* > 0.05) (Table 3). A forest plot of multivariable predictors of ≥50% NRS score reduction at 6 months is provided in Supplementary Materials Figure S2.

### 3.4. Correlation Analysis

Baseline NRS scores positively correlated with NRS score reduction (Pearson r = 0.42, *p* < 0.001). Symptom duration was negatively correlated (Spearman ρ = −0.19, *p* = 0.004) and EPR intervention positively (Spearman ρ = 0.22, *p* = 0.001). Age (r = −0.08, *p* = 0.221) and BMI (r = 0.05, *p* = 0.456) showed no significant correlations (Table 4).

### 3.5. Sensitivity Analysis

Findings were consistent in (i) a model additionally adjusting any covariate with residual imbalance (post-match SMD ≥ 0.10; none materially changed estimates) and (ii) the ODI-subset model including baseline ODI as a covariate. Effect estimates for EPR remained directionally similar and within approximately ±10% of the primary model (Table 5).

## 4. Discussion

This propensity score-matched retrospective cohort study showed that EPR offered significantly better pain relief than PRF in patients with refractory PS. EPR led to lower NRS scores at both 3 and 6 months, with clinically meaningful differences. Additionally, the proportion of patients experiencing a ≥50% reduction in pain was higher with EPR than with PRF at 6 months. Multivariable regression analysis reinforced EPR as a strong independent predictor of successful pain reduction (OR = 2.15). Furthermore, a higher baseline pain score and shorter symptom duration also emerged as significant predictors of treatment effectiveness. Collectively, these findings underline the potential advantages of EPR in achieving effective pain control for refractory PS patients with anatomical entrapment [4,7,11]. The observed between-group differences in NRS scores exceeded the minimal clinically important difference (MCID), previously reported to be ~0.5 points in chronic musculoskeletal pain [12]. This supports the clinical relevance of EPR superiority beyond statistical significance.

Despite higher complication rates with EPR, most events were mild and self-limiting. Patient satisfaction remained higher in the EPR cohort, indicating that patients prioritize effective symptom control even at the cost of manageable risks. While EPR showed a trend toward higher satisfaction and fewer reinterventions, the increased complication rate highlights the importance of careful patient selection and counseling about potential risks. Kaplan–Meier survival analysis for reintervention indicated a non-significant trend favoring EPR (*p* = 0.12). Although the difference did not reach statistical significance, the median time to reintervention was not reached in either group, suggesting potentially longer effectiveness in EPR.

Clinically, our findings are significant as they inform treatment decisions for PS, a notoriously challenging condition often misdiagnosed because of overlapping symptoms with lumbar radiculopathy. The greater pain relief achieved by EPR may lead to an improved overall quality of life for patients, increased mobility, and reduced healthcare use in cases of chronic, refractory pain. Additionally, recognizing factors such as a higher baseline NRS score and shorter symptom duration as predictors of treatment success can help clinicians select suitable patients and customize treatment plans accordingly. While EPR showed a trend toward higher satisfaction and fewer reinterventions, the increased complication rate highlights the importance of careful patient selection and counseling about potential risks. Therefore, these insights can guide shared decision-making between clinicians and patients, weighing the risks and benefits of surgical intervention vs. minimally invasive PRF [11,14].

Another key finding of this study relates to the safety comparison between the two procedures. Although EPR provided better pain relief, it was associated with a higher complication rate (7.8%) compared to PRF (1.7%). Specifically, complications included infections and nerve irritations with EPR, although these were rare. Interestingly, despite the higher complication rate, patients undergoing EPR reported higher satisfaction levels. Additionally, the reintervention rate was lower in the EPR group (6% vs. 12%), though this difference was not statistically significant. This suggests that patients prioritize significant pain relief over procedural risks associated with more invasive treatments, provided they are well-informed. Therefore, clinicians must carefully weigh the procedural risks and benefits, taking into account individual patient preferences and clinical features when recommending either procedure [4,11,14].

Compared to previous unmatched studies reporting pain relief of 50–70% for PRF and 80–90% for EPR, our results align well with existing evidence but offer greater reliability due to rigorous propensity matching [4,11,12]. Unlike smaller, uncontrolled studies that are vulnerable to significant confounding, our matched design provides a more accurate reflection of actual treatment differences. Larionov et al. [7] emphasized the anatomical features of PS that may explain why interventions, such as EPR targeting structural compression, tend to produce better outcomes. Similarly, Vij et al. [11] highlighted surgical options as effective but associated with higher risks of complications. Our findings, therefore, support and expand upon these previous observations, confirming both the efficacy advantage and safety concerns of EPR compared with PRF, especially in cases with confirmed anatomical refractory issues.

Recent developments in minimally invasive and regenerative approaches offer additional therapeutic avenues for refractory PS. Emerging therapies, including ultrasound-guided platelet-rich plasma (PRP) injections and dry needling, have shown promise in preliminary trials [15,16,17]. These regenerative methods focus on facilitating tissue healing and neuromodulation, which can potentially improve procedural outcomes. Similarly, recent studies have supported the combination of advanced imaging techniques, such as high-resolution ultrasound or MRI-guided interventions, to enhance procedural precision and therapeutic effectiveness [13,18]. Furthermore, adjunctive physiotherapy approaches, specifically sciatic nerve mobilization and piriformis muscle release, may complement procedural interventions and form part of a multimodal pain management strategy [19]. Broader PT modalities (e.g., SIS, TECAR therapy, ESWT, Deep Oscillation) were not used in this study but warrant exploration in future research to further standardize rehabilitation and reduce confounding from co-interventions. A recent bibliometric analysis also notes an increase in research focusing on improving diagnostic accuracy and broadening the spectrum of PS treatments [20]. Integrating these novel approaches with established techniques, such as PRF and EPR, could optimize patient outcomes and provide tailored treatment strategies. Our findings align with a case report on PRF for refractory PS showing successful pain relief but align more closely with endoscopic release studies demonstrating superior decompression in deep gluteal syndrome, such as the systematic review by Kay et al., reporting pain improvement in all reviewed studies at a mean 23-month follow-up for surgical management, including endoscopic procedures [6]. This study has several limitations. First, the retrospective design inherently introduces selection bias and limits causal inferences, despite the use of robust propensity score matching. Second, data from a single center may affect generalizability, as results could vary depending on procedural expertise and institutional protocols. Moreover, although the sample size after matching was sufficient for primary outcomes, it may have limited statistical power for secondary outcomes such as satisfaction and reintervention rates. Third, incomplete ODI data restricts comprehensive functional assessments and the interpretation of improvements in disability and daily functioning. The relatively short follow-up period of 6 months may overlook late complications or pain recurrence, highlighting the need for longer follow-up studies. Therefore, prospective multicenter trials with larger cohorts and standardized outcome assessments are necessary to further validate these findings [2,11]. Finally, clinician-level factors (operator experience, procedure volumes) were not available and may influence safety and effectiveness. Additionally, unmeasured confounders such as operator experience and procedure volume may influence outcomes, as these data were not captured.

## 5. Conclusions

In conclusion, EPR offers significantly superior pain relief compared to PRF for refractory piriformis syndrome, although it carries a higher complication risk. Therefore, PRF should initially be considered due to its favorable safety profile, reserving EPR for cases with clear anatomical nerve entrapment or when PRF treatment is unsuccessful. Future prospective studies should also evaluate cost-effectiveness to guide resource allocation in refractory PS management.

## Figures and Tables

**Table 3:** Multivariate logistic regression for ≥ 50% NRS score reduction at 6 months (*n* = 230).

Variable	OR	95% CI	*p*-Value
Intervention (EPR vs. PRF)	2.15	1.12–4.13	0.021
Age (per years)	0.99	0.97–1.02	0.612
Sex (F vs. M)	1.14	0.62–2.09	0.674
BMI (per kg/m^2^)	1.03	0.96–1.11	0.401
Symptom duration (per years)	0.87	0.76–0.99	0.038
Baseline NRS (per unit)	1.32	1.08–1.61	0.007
Diabetes (Yes vs. No)	0.82	0.39–1.73	0.601

NRS score reduction *n* = percentage decrease in NRS score at 6 months. NRS: Numeric Rating Scale; BMI: body mass index; EPR: endoscopic piriformis release; PRF: pulsed radiofrequency. Pearson for continuous variables; Spearman for categorical/ordinal. Clinical significance was interpreted in reference to MCID of ~0.5 points for NRS scores.

**Table 4:** Correlation analysis with NRS score reduction at 6 months.

Variable	Correlation Coefficient	*p*-Value
Baseline NRS	r = 0.42 (Pearson)	<0.001
Symptom duration	ρ = −0.19 (Spearman)	0.004
Age	r = −0.06 (Pearson)	0.372
BMI	r = 0.04 (Pearson)	0.551
Intervention (EPR vs. PRF)	ρ = 0.22 (Spearman)	0.001

RS reduction = percentage decrease in NRS score at 6 months. NRS: Numeric Rating Scale; BMI: body mass index; EPR: endoscopic piriformis release; PRF: pulsed radiofrequency. Pearson for continuous variables; Spearman for categorical/ordinal.

**Table 5:** Sensitivity analyses.

Model	Key Covariates	EPR Effect (Direction vs. Primary)
Primary model	Age, sex, BMI, symptom duration, baseline NRS, diabetes, hypertension	References
Residual-imbalance model	Primary + any covariate with post-match SMD ≥ 0.10	Similar
ODI-subset model	Primary + baseline ODI (subset *n* = 90)	Similar

## Data Availability

The datasets analyzed are available from the corresponding author upon reasonable request.

## Supplementary Materials

The following supporting information can be downloaded at: https://www.mdpi.com/article/10.3390/jcm14165908/s1, Figure S1: A love plot of SMD; Figure S2: A forest plot of multivariable predictor.

## Abbreviations

PS	Piriformis Syndrome
PRF	Pulsed Radiofrequency
EPR	Endoscopic Piriformis Release
PSM	Propensity Score Matching
NRS	Numeric Rating Scale (0–10 scale for pain)
ODI	Oswestry Disability Index
BMI	Body Mass Index
FAIR test	Flexion, Adduction, and Internal Rotation Test
MCID	Minimal Clinically Important Difference
SD	Standard Deviation
OR	Odds Ratio
CI	Confidence Interval
NSAIDs	Non-Steroidal Anti-Inflammatory Drugs
PRP	Platelet-Rich Plasma
MRI	Magnetic Resonance Imaging
