# Supplementary material for: Comparison of Pulsed Radiofrequency and Endoscopic Piriformis Release for Refractory Piriformis Syndrome: A Propensity Score-Matched Retrospective Cohort Study
Source: J Clin Med. 2025 Aug 21;14(16):5908. doi: 10.3390/jcm14165908 (PMC12387164; doi:10.3390/jcm14165908)
Supplement: Supplementary file 1 [file jcm-14-05908-s001.zip › jcm-3802306-supplementary.pdf]

**Supplementary Materials**

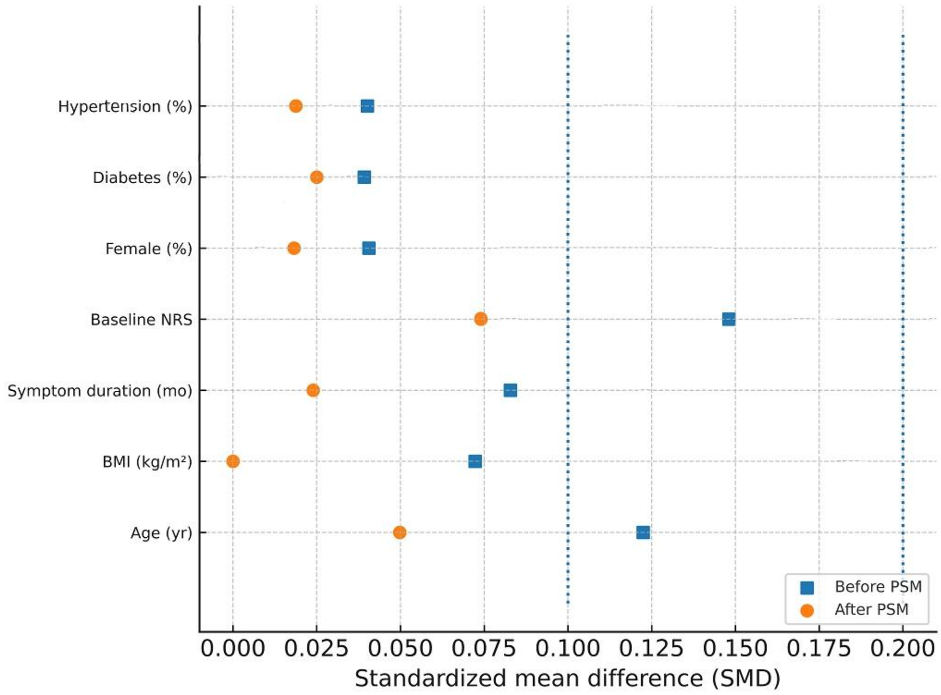


**SFig.1. A love plot of SMD**

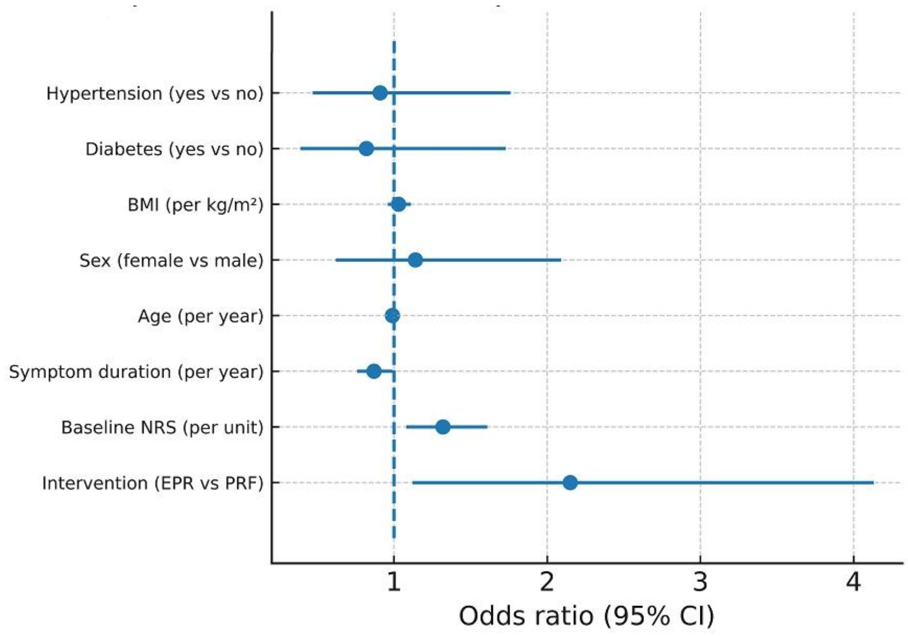


**SFig.2. A forest plot of multivariable predictors**
